# Trends in the Prevalence of Tooth Loss Due to Dental Caries During Periods of Dental Health Insurance Expansion in Korea

**DOI:** 10.3290/j.ohpd.c_2749

**Published:** 2026-09-29

**Authors:** Jin-Sun Choi, Deuk-Sang Ma

**Affiliations:** a Jin-Sun Choi Researcher, Department of Dental Hygiene, College of Dentistry, Kangwon National University, Gangwon, South Korea; Research Institute of Oral Science, Kangwon National University, Gangwon, South Korea; Conceptualised and designed the study, performed data analysis, drafted the manuscript, and read and approved the final version of the manuscript.; b Deuk-Sang Ma Researcher, Department of Preventive and Public Health Dentistry, College of Dentistry, Kangwon National University, Gangwon, South Korea; Research Institute of Oral Science, Kangwon National University, Gangwon, South Korea. Contributed to study design, interpretation of data, and critical revision of the manuscript, and read and approved the final version of the manuscript.

**Keywords:** dental caries, health insurance, Korea National Health and Nutrition Examination Survey (KNHANES), tooth loss

## Abstract

**Purpose:**

This study aimed to examine national trends in the prevalence of tooth loss due to dental caries among Korean adults across periods of dental health insurance expansion.

**Materials and Methods:**

Using data from the Korea National Health and Nutrition Examination Survey for 2007–2009 (pre-policy period) and 2016–2018 (post-policy period), this study examined changes in the prevalence of tooth loss due to dental caries among adults aged 19 years and older. A complex sample logistic regression analysis was conducted, adjusting for covariates such as sex, age, region, income level, toothbrushing frequency, and dental checkup history.

**Results:**

The prevalence of tooth loss due to dental caries was lower in 2016–2018 compared with 2007–2009. After adjustment for covariates, the odds of caries-related tooth loss were significantly lower in the later period (*P* < 0.001). However, a higher prevalence persisted among older adults, individuals with lower income, and rural residents.

**Conclusion:**

This study identified a declining trend in caries-related tooth loss during periods of dental health insurance expansion in Korea. Despite overall improvements, socioeconomic and regional disparities remained evident, indicating the need for targeted strategies to address oral health inequalities among vulnerable populations.

Tooth loss presents significant challenges in daily life, particularly in relation to dietary habits and social interactions.^23^ A reduction in the number of teeth compromises mastication, leading to inefficient food breakdown and adverse effects on swallowing.^21^ In addition, tooth loss has been associated with various systemic conditions, including cardiovascular disease, thereby complicating overall health status.^20^ Consequently, preventing tooth loss and maintaining functional dentition are important for both physical health and quality of life.

Health insurance policies have been shown to play an important role in improving access to dental care and reducing disparities in oral healthcare utilisation. Studies from Canada indicate that individuals with dental insurance are more likely to use oral health services than those without coverage.^18^ In Peru, long-term insurance enrollment has been associated with greater access to dental services compared with shorter coverage periods.^7^ Similarly, in the United States, expansions in public insurance programs have been linked to increased utilisation of preventive dental care among low-income populations.^3,15^ Collectively, these findings suggest that health insurance coverage is closely related to patterns of dental care utilisation.

South Korea operates a mandatory National Health Insurance system based on a cost-sharing structure, in which the National Health Insurance Service subsidises healthcare expenses to reduce individuals’ financial burden.^19^ Since 2009, dental health insurance coverage has been progressively expanded as part of national efforts to improve oral health. Major policy changes included the introduction of sealant coverage for children aged 6–14 years in 2009, denture coverage for adults aged 75 years and older in 2012, scaling benefits for adults aged 20 years and older in 2013, and dental implant coverage in 2014.^17,19^ These policy changes substantially altered the landscape of dental care access in Korea, particularly with respect to preventive services such as scaling.^8^

Despite these policy developments, previous Korean studies have primarily focused on changes in dental service utilisation or cost-related outcomes following insurance expansion. In contrast, relatively little attention has been paid to population-level changes in oral health outcomes, particularly tooth loss due to dental caries. Moreover, most existing studies have relied on single-year cross-sectional analyses, limiting their ability to examine changes across policy-relevant periods using nationally representative data.

Therefore, the present study aimed to examine national trends in the prevalence of tooth loss due to dental caries among Korean adults across periods of dental health insurance expansion, using data from the Korea National Health and Nutrition Examination Survey. By comparing two policy-relevant periods and accounting for the complex survey design, this study seeks to provide descriptive evidence on changes in caries-related tooth loss and to inform discussions on future strategies to reduce oral health inequalities.

## MATERIALS AND METHODS

### Study Participants

This study employed a cross-sectional comparative design using pooled data from the Korea National Health and Nutrition Examination Survey (KNHANES), a nationally representative survey conducted to assess the health and nutritional status of the Korean population. The dataset was obtained with official approval from the KNHANES website.^11^

KNHANES uses a complex, stratified, multistage probability sampling design to ensure representativeness and accurate population-level estimates.^11^ Oral examinations were conducted by trained dentists at mobile examination centres using standardised protocols. Examiner reliability was periodically assessed through training and calibration procedures, including the calculation of kappa statistics.^11^

To examine population-level trends in caries-related tooth loss across policy-relevant periods, data from two survey cycles were compared: 2007–2009 and 2016–2018. The earlier period represents a baseline prior to major dental insurance expansions, while the latter corresponds to a period after the stabilisation of key dental coverage policies. The intermediate years (2010–2015) were considered a transitional phase and were therefore excluded to allow clearer period comparisons.

The study population included adults aged 19 years and older who completed oral examinations. The final analytic sample consisted of 16,452 participants from 2007 to 2009 and 12,730 participants from 2016 to 2018. Cases with missing data in the outcome variable or key covariates were excluded, and analyses were conducted using participants with complete data only.

### Variables and Statistical Analysis

The outcome variable was tooth loss due to dental caries, categorised as a binary variable (Yes/No) based on clinical oral examination data. Covariates were selected a priori based on previous literature and included sex, age group (19–39, 40–64, ≥ 65 years), residential region (urban or rural), household income level (quartiles 1–4), tooth brushing frequency (≥ 2 times/day or < 2 times/day), and dental checkup history within the past year (Yes/No).

Weighted prevalence estimates were calculated to account for the complex survey design. Differences in prevalence across covariates and survey periods were assessed using design-based Chi-square tests. Complex sample logistic regression analyses were performed to estimate crude and adjusted odds ratios (ORs) with 95% confidence intervals (CIs), adjusting for potential confounders. Crude ORs for covariates were estimated separately for each variable within each survey period. Adjusted ORs for covariates were estimated separately for each survey period using multivariable logistic regression models including sex, age, residential region, household income level, toothbrushing frequency, and dental checkup history within the past year. The OR for the survey period was estimated using the pooled dataset, and the adjusted OR was estimated after adjustment for all covariates.

All analyses incorporated KNHANES sampling weights, strata, and primary sampling units to obtain nationally representative estimates. A two-sided significance level of 0.05 was applied. Statistical analyses were conducted using Stata Statistical Software (Release 18; StataCorp LLC, College Station, TX, USA).

### Ethics Approval

The KNHANES was approved by the Institutional Review Board of the Korea Centres for Disease Control and Prevention (Approval Nos: 2010-02CON-21-C, 2012-01EXP-01-2C, and 2018-01-03-P-A). Data from 2016 and 2017 were exempt from additional IRB review as part of national public welfare research.^16^ This study was granted exemption from further ethical review for secondary data analysis by the Institutional Review Board of Gangneung-Wonju National University (GWNUIRB-R2025-11).

## RESULTS

### General Characteristics of Participants

Table 1 summarises the general characteristics of the study participants by survey period.

**Table 1:** General characteristics of study participants

Category	Pre-policy period (2007–2009) – N	Pre-policy period (%)	Post-policy period (2016–2018) – N	Post-policy period (%)
**Total**	16,452	100.0	12,730	100.0
**Sex**				
Male	6,988	42.5	5,558	43.7
Female	9,464	57.5	7,172	56.3
**Age group**				
19–39 years	5,501	33.4	3,611	28.4
40–64 years	7,299	44.4	5,981	47.0
≥ 65 years	3,652	22.2	3,138	24.7
**Region**				
Urban	12,079	73.4	10,476	82.3
Rural	4,373	26.6	2,254	17.7
**Household income**				
Lowest (1)	3,528	21.4	2,424	19.0
2	4,097	24.9	3,107	24.4
3	4,380	26.6	3,535	27.8
Highest (4)	4,447	27.0	3,664	28.8
**Brushing frequency**				
< 2 times per day	2,362	14.4	1,191	9.4
≥ 2 times per day	14,090	85.6	11,539	90.6
**Dental checkup in the past year**				
No	11,847	72.0	8,176	64.2
Yes	4,605	28.0	4,554	35.8


Compared with the earlier period, the proportion of participants aged 65 years and older increased from 22.2% to 24.7% in 2016–2018. The proportion of urban residents increased from 73.4% to 82.3%, while the proportion of rural residents decreased from 26.6% to 17.7%. Health-related behaviours also differed across periods: the proportion of individuals brushing their teeth at least twice daily increased from 85.6% to 90.6%, and the proportion reporting an oral examination within the past year increased from 28.0% to 35.8% (Table 1).

### Prevalence of Tooth Loss Due to Dental Caries by Covariates

Across both survey periods, females consistently exhibited a higher prevalence of caries-related tooth loss than males. In 2007–2009, the prevalence was 43.5% among females and 37.0% among males; in 2016–2018, the prevalence decreased in both groups but remained higher among females (38.0%) than among males (35.0%) (Table 2).

**Table 2:** Rate of tooth loss due to dental caries by year

Variables	N	Yes	wt(%)
Total	29,182	12,577	39.4
Pre-policy period (2007–2009)	16,452	7,827	40.3
Post-policy period (2016–2018)	12,730	4,750	36.8


Age-related gradients were pronounced in both periods. The highest prevalence was observed among adults aged 65 years and older (77.3% in 2007–2009 and 63.9% in 2016–2018). Among adults aged 40–64 years, the prevalence declined from 49.6% to 36.7%, whereas among younger adults aged 19–39 years, prevalence remained relatively low in both periods (19.3% and 14.0%, respectively).

Rural residents consistently showed a higher prevalence of tooth loss than urban residents (pre-policy: 51.1% vs 37.6%; later period: 48.2% vs 34.6%). A clear socioeconomic gradient was also observed: individuals in the lowest income quartile reported the highest prevalence (59.1% in 2007–2009 and 57.2% in 2016–2018), while those in the highest-income quartile showed substantially lower prevalence (32.0% and 26.9%, respectively).

Behavioural factors were similarly associated with tooth loss prevalence. Participants who brushed their teeth fewer than two times per day had a higher prevalence than those brushing at least twice daily in both periods. In addition, individuals who had not undergone an oral examination within the past year consistently exhibited a higher prevalence than those who had (Table 3).

**Table 3:** Association between covariates and tooth loss due to dental caries

Variables	Pre-policy period (2007–2009)				Post-policy period (2016–2018)			
	N	Yes	wt(%)	*P* value	N	Yes	wt(%)	*P *value
**Total**	16,452	7,827	40.3		12,730	4,750	36.8	
**Sex**								
Male	6,988	3,149	37.0	*P* < 0.001	5,558	1,990	35.0	*P* < 0.01
Female	9,464	4,678	43.5		7,172	2,760	38.0	
**Age**								
19–39 years	5,501	1,086	19.3	*P* < 0.001	3,611	526	14.0	*P* < 0.001
40–64 years	7,299	3,832	49.6	*P* < 0.001	5,981	2,234	36.7	
65 years and older	3,652	2,909	77.3	*P* < 0.001	3,138	1,990	63.9	
**Region**								
Urban	12,079	5,121	37.6	*P* < 0.001	10,476	3,673	34.6	*P* < 0.001
Rural	4,373	2,706	51.1	*P* < 0.001	2,254	1,077	48.2	
**Household income**								
1 (Lowest)	3,528	2,432	59.1	*P* < 0.001	2,424	1,380	57.2	*P* < 0.001
2	4,097	2,069	43.3		3,107	1,246	39.8	
3	4,380	1,741	35.6		3,535	1,112	30.9	
4 (Highest)	4,447	1,585	32.0		3,664	1,012	26.9	
**Brushing frequency**								
Less than twice a day	2,362	1,441	51.4	*P* < 0.001	1,191	594	49.3	*P *< 0.001
Twice or more a day	14,090	6,386	38.7		11,539	4,156	35.5	
**Dental checkup in the past year**								
No	11,847	5,864	41.4	*P* < 0.001	8,176	3,250	39.5	*P *< 0.001
Yes	4,605	1,963	37.8		4,554	1,500	36.8	


### Analysis of the Association Between Covariates and Tooth Loss due to Dental Caries

Table 4 presents crude and adjusted odds ratios for caries-related tooth loss. After adjustment for sociodemographic and behavioural covariates, the odds of tooth loss due to dental caries were significantly lower in 2016–2018 compared with 2007–2009 (adjusted OR = 0.60, 95% CI: 0.57–0.64), corresponding to approximately 40% lower odds in the later period.

**Table 4:** Association between covariates and tooth loss due to dental caries

Variables	Crude OR (95% CI)		Adjusted OR (95% CI)	
	Pre-policy period (2007–2009)	Post-policy period (2016–2018)	Pre-policy period (2007–2009)	Post-policy period (2016–2018)
**Total**	1.00	0.86 (0.81-0.91)***	1.00	0.60 (0.57–0.64)***
**Gender**				
Male (Ref.)	1.00	1.00	1.00	1.00
Female	1.31 (1.21–1.42)^***^	1.14 (1.04–1.24)^**^	1.26 (1.15–1.37)^***^	1.09 (0.99–1.19)
**Age**				
19–39 years (Ref.)	1.00	1.00	1.00	1.00
40–64 years	4.11 (3.72–4.55)^***^	3.57 (3.16–4.04)^***^	4.05 (3.66–4.48)^***^	3.56 (3.15–4.04)^***^
**65 years and older**	14.24 (12.51–16.22)^***^	10.90 (9.51–12.49)^***^	11.16 (9.70–12.83)^***^	8.59 (7.43–9.94)^***^
Region				
Urban (Ref.)	1.00	1.00	1.00	1.00
Rural	1.76 (1.61–1.97)^***^	1.76 (1.57–1.97)^***^	1.32 (1.19–1.46)^***^	1.38 (1.22–1.56)^***^
**Household income**				
4 (Highest) (Ref.)	1.00	1.00	1.00	1.00
3	1.18 (1.06–1.31)^**^	1.22 (1.08–1.37)^**^	1.24 (1.10–1.39)^***^	1.16 (1.03–1.31)^**^
2	1.63 (1.46–1.82)^***^	1.80 (1.60–2.02)^***^	1.47 (1.30–1.66)^***^	1.39 (1.22–1.58)^***^
1 (Lowest)	3.08 (2.73–3.47)^***^	3.64 (3.21–4.13)^***^	1.69 (1.47–1.93)^***^	1.73 (1.49–2.00)^***^
**Brushing frequency**				
Twice or more a day (Ref.)	1.00	1.00	1.00	1.00
Less than twice a day	1.68 (1.50–1.88)^***^	1.76 (1.53–2.03)^***^	1.17 (1.02–1.34)^*^	1.11 (0.95–1.30)^NS^
**Dental checkup in the past year**				
Yes (Ref.)	1.00	1.00	1.00	1.00
No	1.16 (1.07–1.27)*	1.38 (1.27–1.52)***	1.00 (0.90–1.11)NS	1.19 (1.08–1.31)^**^
Significance levels: NS (*P* > 0.05), * P < 0.05, ** P < 0.01, *** *P* < 0.001. OR, odds ratio; CI, confidence interval. Crude ORs were estimated separately for each variable within each survey period. Adjusted ORs for covariates were estimated separately for each survey period using multivariable logistic regression models including sex, age, region, household income, toothbrushing frequency, and dental checkup in the past year. The OR for the survey period (Total) was estimated using the pooled dataset; the adjusted OR was additionally adjusted for all covariates listed above. NS, not significant.				

Across both periods, older age, rural residence, and lower household income were strongly associated with higher odds of caries-related tooth loss, with particularly elevated odds among adults aged 65 years and older and those in the lowest income quartile. In the post-policy period, not having a dental checkup in the past year was significantly associated with higher odds of tooth loss due to dental caries (Table 4).

## DISCUSSION

This study analysed temporal changes in the prevalence of tooth loss due to dental caries and associated factors using nationally representative data from the Korea National Health and Nutrition Examination Survey (KNHANES) collected between 2007 and 2018. The findings indicate a general decline in the prevalence of caries-related tooth loss during the post-policy period (2016–2018) compared with the pre-policy period (2007–2009). However, significant differences in prevalence persisted according to sex, age, region, household income, and oral health behaviours.

The overall prevalence of dental caries-related tooth loss decreased from 40.3% in the pre-policy period to 36.8% in the post-policy period. After adjusting for age, sex, income level, region, and oral health behaviours, the likelihood of experiencing tooth loss due to dental caries was significantly lower in the post-policy period. Although this study does not establish a causal relationship between health insurance expansion and tooth loss outcomes, the observed decline coincided with a period characterised by strengthened oral health policies, improved accessibility to dental care, and increased utilisation of preventive dental services.

These findings are consistent with previous studies conducted in other countries, which have reported associations between expanded public dental coverage and improved oral health outcomes. For example, studies in the United States have shown that Medicaid dental coverage expansion increased access to dental care among low-income adults, although its association with tooth loss has been complex and may partly reflect previously unmet treatment needs.^24^ Similarly, analyses of data from the Canadian Community Health Survey showed that government-funded dental insurance for older adults improved access to regular dental care and reduced financial barriers.^27^ Other studies have reported that comprehensive dental coverage reduced unmet dental care needs and improved oral health among low-income populations.^15^ The Affordable Care Act in the United States also aimed to enhance dental care accessibility for vulnerable populations, reinforcing the notion that improved insurance coverage may be linked to better oral health outcomes.^3^ However, differences in healthcare system structures, eligibility criteria, and benefit designs limit direct comparability with the Korean context, and these international findings should be interpreted as contextual references rather than direct evidence of policy effectiveness in Korea.

In South Korea, previous studies have reported that the inclusion of dental implants under the National Health Insurance was associated with a significant increase in the number of patients receiving implant treatment, suggesting improved access to restorative dental care among older and socioeconomically vulnerable populations.^13^ While implant coverage itself does not directly prevent dental caries, it may reflect broader improvements in dental service utilisation following insurance expansion.

Despite the overall decline, approximately 37% of the population still experienced tooth loss due to dental caries in the post-policy period. Older adults, particularly those aged 65 years and older, showed the highest prevalence, highlighting persistent vulnerability in this group. Tooth loss in older adults is influenced not only by cumulative caries experience but also by chronic diseases, polypharmacy, and periodontal conditions. These findings emphasise the importance of strengthened oral health management strategies for older adults, including regular dental checkups and preventive care.^12,16^

Socioeconomic disparities were also evident. Individuals in lower-income groups consistently exhibited higher prevalence of tooth loss due to dental caries, with the lowest-income group reporting substantially higher rates than the highest-income group in both policy periods. This gradient underscores the impact of financial constraints on access to and continuity of dental care.^9^ Economic deprivation has been strongly linked to dental diseases, and limited financial resources may delay preventive care, leading to disease progression and eventual tooth loss.^5^ Expanding dental insurance benefits for low-income populations and increasing financial support for preventive dental care remain important public health priorities.

Sex-based differences were observed, with women showing a higher overall prevalence of tooth loss due to dental caries than men. However, the decline in prevalence following policy expansion was more pronounced among women, suggesting differential utilisation or responsiveness to dental services. Previous studies indicate that women generally have higher oral health literacy and more favourable attitudes toward preventive dental care.^25^ Conversely, men tend to utilise dental services less frequently and are more likely to seek care only when symptoms become severe, which may limit the benefits of preventive policies.^10,14^ These findings suggest that sex-specific oral health promotion strategies may be warranted.

Geographic disparities persisted across both periods, with rural residents consistently exhibiting a higher prevalence of tooth loss than urban residents. Although a decline was observed in both groups, barriers such as limited availability of dental professionals, geographic isolation, and transportation challenges likely continue to restrict access to dental care in rural areas.^6^ Financial, geographic, and social barriers remain key challenges to oral healthcare access, particularly in economically disadvantaged regions.^2,4^ Policies aimed at expanding mobile dental services, improving infrastructure, and enhancing preventive coverage in rural areas may help reduce these disparities.

Oral health behaviours played a critical role in maintaining dentition. Individuals who brushed their teeth at least twice per day consistently exhibited lower prevalence of tooth loss due to dental caries, whereas those who brushed less frequently had substantially higher prevalence. These findings support existing evidence emphasising the importance of regular tooth brushing in preventing dental caries and tooth loss.^22^ In addition, regular dental checkups were associated with lower prevalence of tooth loss, consistent with long-term studies demonstrating that adherence to professional oral maintenance reduces cumulative tooth loss over time.^1^ Increasing participation in regular dental examinations through expanded insurance coverage and public health initiatives may further contribute to improved oral health outcomes.

Several limitations should be acknowledged. First, this study was based on a comparison between two independent cross-sectional survey periods rather than longitudinal follow-up data. Therefore, causal inferences regarding the direct effects of dental insurance expansion on tooth loss outcomes cannot be established. In addition, the repeated cross-sectional design limits the ability to disentangle policy-related changes from broader secular trends, such as changes in diet, oral hygiene products, public health campaigns, and healthcare utilisation patterns. Second, tooth loss due to dental caries was assessed based on oral examination data, which may be subject to misclassification bias, particularly in distinguishing caries-related tooth loss from periodontal causes. Third, analyses were conducted using a complete-case approach, which may introduce selection bias if missing data were not random. Finally, the suspension of oral health examinations in the KNHANES during the COVID-19 pandemic limited the availability of post-2019 data, restricting evaluation of longer-term trends following more recent policy changes.

Despite these limitations, this study provides nationally representative evidence of temporal changes in the prevalence of tooth loss due to dental caries in Korea. The observed decline highlights improvements in population oral health indicators during a period of expanded dental insurance coverage and enhanced preventive care access. Continued monitoring of oral health trends and targeted preventive strategies for older adults, rural residents, and low-income populations are essential to reduce persistent disparities and improve national oral health outcomes.

This study identified a declining trend in the prevalence of tooth loss due to dental caries during a period of expanded dental health insurance coverage in Korea. However, older adults, rural residents, and low-income populations continued to experience disproportionately high rates of tooth loss, suggesting persistent barriers to dental care access and affordability. These findings highlight the need for continued monitoring of oral health trends and targeted policy efforts focused on vulnerable populations.

### Acknowledgements 

We would like to thank the Korea Centres for Disease Control and Prevention for providing access to the raw data from the Korea National Health and Nutrition Examination Survey (KNHANES).

#### Conflict of interest

The authors have no conflicts of interest to declare.
